# A computationally efficient dynamic model of human epicardial tissue

**DOI:** 10.1371/journal.pone.0259066

**Published:** 2021-10-26

**Authors:** Niccoló Biasi, Alessandro Tognetti

**Affiliations:** 1 Department of Information Engineering, University of Pisa, Pisa, Italy; 2 Research Centre “E. Piaggio”, University of Pisa, Pisa, Italy; University of Minnesota, UNITED STATES

## Abstract

We present a new phenomenological model of human ventricular epicardial cells and we test its reentry dynamics. The model is derived from the Rogers-McCulloch formulation of the FitzHugh-Nagumo equations and represents the total ionic current divided into three contributions corresponding to the excitatory, recovery and transient outward currents. Our model reproduces the main characteristics of human epicardial tissue, including action potential amplitude and morphology, upstroke velocity, and action potential duration and conduction velocity restitution curves. The reentry dynamics is stable, and the dominant period is about 270 ms, which is comparable to clinical values. The proposed model is the first phenomenological model able to accurately resemble human experimental data by using only 3 state variables and 17 parameters. Indeed, it is more computationally efficient than existing models (i.e., almost two times faster than the minimal ventricular model). Beyond the computational efficiency, the low number of parameters facilitates the process of fitting the model to the experimental data.

## Introduction

Heart disease is a leading cause of death worldwide and causes millions of victims every year. According to the 2019 statistics from the European Society of Cardiology [1], cardiovascular disease (CVD) accounts for 43% of all death in Europe (47% in females and 39% in males). The leading cause of mortality is ischaemic heart disease which accounts for 41% of all CVD deaths. In the majority of these patients, sudden cardiac death is thought to be due to ventricular tachyarrhythmias (i.e., reentrant waves). Mathematical models of the electrical activity of the heart are recognized as important tools in modern cardiac research. First, they are extremely useful in understanding of cardiac pathophysiology, for example, in case of cardiac arrhythmias. Mathematical models could be also employed to simulate clinical recordings (e.g., ECG) under both healthy and pathological conditions. Simulations of such signals are very helpful for the development of new diagnostic tools. The possibilities for doing experimental and clinical studies involving human heart tissues are extremely limited. Moreover, the electrophysiological properties of animal cardiac tissues are different from human cardiac tissue [2–4]. Modelling heart tissue electrical activity requires the use of ionic models reproducing the electrophysiological properties of cardiac cells. Ionic models can be divided in two categories: physiological and phenomenological models. Physiological models aim at describing all the membrane currents that occur in cardiac cells. On the other hand, phenomenological models aim at reproducing the integral characteristics of action potentials (APs) propagation in cardiac tissue. In this paper we present a new phenomenological model of human ventricular epicardial cells and we test its reentry dynamics. To the best of the authors’ knowledge, the proposed model is the first phenomenological model able to accurately resemble human experimental data by using only 3 state variables and 17 parameters. Therefore, our model is more computationally efficient than existing ones and the low number of parameters facilitates the process of fitting the model to the experimental data.

Several physiological model based on human data have been developed previously. The first developed ionic model for human epicardial cells is the Priebe–Beuckelmann (PB) model [5]. The PB model was derived by the phase 2 Luo-Rudy (LR) model [6], originally verified with guinea pig ventricular cells. However, it is partially based on human data (i.e. only five of the ten ionic currents are based on human experimental data), and can reproduce several properties of healthy and failing myocytes. Nevertheless, the action potential duration (APD) is longer than typical values recorded in human ventricular tissue. The PB model consists of 22 variables, but a simplified 6-variable version was developed by Bernus et al. [7]. Subsequently, the Ten Tusscher–Noble–Noble–Panfilov (TNNP) model [8] was developed. The TNNP model is based on more recent human experimental data and consists of 17 variables. An improved version of the model, including a more extensive description of intracellular calcium dynamics, was published in 2006 [9]. Moreover, as for the PB model, a simplified version with 9 variables was developed [10]. A third physiological model is the Iyer–Mazhari–Winslow (IMW) model [11]. Differently from the PB and TNNP models, the IMW model is notable for using Markov chains to model the dynamics of ion channel gates, instead of Hodgkin–Huxley-type equations. Consequently, the IMW model consists of a very large number of variables (i.e., 67). More recently, other two physiological models were developed. First, the Grandi-Pasqualini-Bers (GPB) model [12] consists of 38 state variables, and provides a more detailed description of intracellular calcium dynamics. Second, the O’Hara-Virág-Varró-Rudy (OVVR) model [13] consists of 41 state variable. Its main limitation is the reduced conduction velocity (CV) when compared to experimental data. The tissue-level characteristics of both the GPB and the OVVR models were studied in [14].

Physiological models are very useful in understanding pathophysiology of cardiac diseases, however their applicability in tissue-level simulations can be limited by the computational complexity. On the contrary, phenomenological models have a lower number of variables, but they cannot be used to describe ionic current changes. Nevertheless, they are powerful tools for computationally efficient two- and three-dimensional tissue-level simulations. Indeed, phenomenological models also relies on a smaller number of parameters facilitating accurate tuning of the model to reproduce the desired behaviour. The first phenomenological model based on human experimental data is the minimal ventricular (MV) model proposed by Bueno-Orovio et al. [15]. The MV model is based on only 4 variables, and is directly derived from the three-variable model proposed by Fenton and Karma [16]. In particular, the fourth variable was added to reproduce accurate AP shapes. The MV model can reproduce the main tissue level characteristics of epicardial, endocardial and midmyocardial cells. Subsequently, Peñaranda et al. [17] proposed a new phenomenological model, which was still based on the Fenton-Karma model. The authors added a fourth current describing the transient outward current, which was modelled with two additional variables. Therefore, the 4-current model consists in total of 5 variables, and correctly reproduces restitution properties and AP morphologies.

To build our model we started from the FitzHugh-Nagumo (FHN) equations [18] in the Rogers-McCulloch formulation [19] because it is simple and computationally efficient. Note that the FHN model has only two state variables. First, we introduced a shape factor to obtain accurate AP morphologies. Then, we modified the definition of the timescale of the recovery variable to fit the experimental restitution properties. Finally, we added a third state variable to describe the transient outward current, and thus the AP notch. As the MV model, our model can reproduce the main tissue-level characteristics of epicardial cells, such as AP amplitudes and shapes, upstroke velocities, and APD and CV restitution curves. The reentry dynamics of our model shows a stable spiral wave with a dominant period in the clinical range for ventricular tachycardia. Furthermore, our model proved to be almost two times faster than the MV model.

## Materials and methods

### Model formulation

We developed a novel computationally efficient model for human ventricular epicardial cells reproducing the currently available human experimental data [20–25], also used for the development of the MV model [15]. Our model is based on the Rogers-McCulloch formulation of the FHN model [19]. In our model the trasmembrane current is represented as the sum of three contributions:

$$\begin{array}{c} I_{ion}=gk(I_{exc}+I_{rec}+I_{to}) \end{array}$$ (1)

where *I*_*exc*_, *I*_*rec*_, *I*_*to*_ are the excitatory, recovery, and transient outward currents, respectively. With respect to the Rogers-McCulloch formulation, we added the description of transient outward current *I*_*to*_, and we introduced the shape factor *g* to accurately fit the AP morphology. *k* is a parameter defining the timescale of the model. The definitions of excitatory and recovery currents is unchanged:

$$\begin{array}{c} I_{exc}=c_{1}(V_{M}-B)(a-\frac{V_{M}-B}{A})(1-\frac{V_{M}-B}{A}) \end{array}$$ (2)

$$\begin{array}{c} I_{rec}=c_{2}u(V_{M}-B) \end{array}$$ (3)

where A and B represent the AP amplitude and the resting potential, respectively. The parameter *a* defines the activation threshold of the excitatory current (i.e., *I*_*exc*_ becomes negative only when *V*_*M*_ > *aA* + *B*). *c*_1_ and *c*_2_ are two model parameters determining the intensity of excitatory and recovery currents. Finally, *u* is the recovery variable of the model. According to [19], it is defined by the following differential equation:

$$\begin{array}{c} \frac{\partial u}{\partial t}=ke(\frac{V_{M}-B}{A}-u) \end{array}$$ (4)

where *e* is the inverse of the time constant of the variable. We used a novel formulation for defining *e*:

$$\begin{array}{c} e=\{\begin{array}{c} e_{1}\;if\;\frac{\partial u}{\partial t}\geq 0\\ e_{2}\;if\;\frac{\partial u}{\partial t}<0 \end{array} \end{array}$$ (5)

Such formulation allows us to independently control the APD through *e*_1_ and the refractoriness of the model by means of *e*_2_. Note that the value of *e* only depends on the sign of the derivative of *u*, which does not depend on *e*. Therefore, an initial value for *e* is not needed. FHN-like models cannot reproduce accurate AP shapes. Indeed, such models are characterized by a relationship between upstroke and recovery velocity. This flaw is due to the inability of modulating the ionic current during the different phases of the action potential. We solved this drawback by means of the shape factor *g* we introduced in Eq 1, avoiding introduction of additional variables. In particular, *g* should have an high value during the AP upstroke and should decay in the last phases of the AP. A possible approach is to define *g* as a function of *u*:

$$\begin{array}{c} g=(\gamma_{0}+\gamma_{1}u)\frac{\left(-tanh(\alpha (u-\theta_{u}))+1\right)}{2}+g_{0} \end{array}$$ (6)

This formulation of *g* allowed us to reproduce accurate AP shapes, and also to fit the restitution properties of the model to the currently available experimental data. Moreover, the suitability of the hyperbolic tangent function in reproducing cardiac dynamics has been already recognized [15–17]. In order to maintain the same balance between excitatory and recovery current during upstroke, we defined *e*_1_ as follows:

$$\begin{array}{c} e_{1}=\{\begin{array}{cc} ge_{1}^{0}\; & if\;upstroke\\ e_{1}^{0}\; & otherwise \end{array} \end{array}$$ (7)

The numerical implementation of this equation is trivial and will be discussed later. Finally, the transient outward current is defined through an additional variable *w*:

$$\begin{array}{c} I_{to}=c_{3}w(V_{M}-B)s_{u} \end{array}$$ (8)

where *c*_3_ is a model parameter analogous to *c*_1_ and *c*_2_. *s*_*u*_ is a function of *u* with two objectives. First, it dissolves the transient outward current in the last phases of the AP. Second, it reduces the intensity of *I*_*to*_ when the cardiac tissue is in a refractive state. Even if the restitution properties of AP notch has not been widely investigated yet, a reduction of transient outward current at small diastolic intervals (DIs) has been found experimentally [20, 23]. Furthermore, also complex physiological models show vanishing of the AP notch at small DIs [5, 8]. *s*_*u*_ is defined as:

$$\begin{array}{c} s_{u}=\frac{{\left(u_{M}-u\right)}^{2}}{u_{M}^{2}} \end{array}$$ (9)

where *u*_*M*_ is a model parameter whose value should be higher than the maximum value of *u*. We selected this function to reproduce experimental results regarding AP morphology (e.g., plateau voltage). The third state variable *w* of our model is defined according the following differential equation:

$$\begin{array}{c} \frac{\partial w}{\partial t}=ke_{w}(\frac{V_{M}-B}{A}-d_{w}w) \end{array}$$ (10)

*d*_*w*_ determines the asymptotic value of the variable *w*. In particular, a lower value of *d*_*w*_ implies higher values of *w*, thus a stronger transient outward current. To correctly reduce the value of *w* and *I*_*to*_ when *u* increases, we defined *d*_*w*_ on the basis of *s*_*u*_:

$$\begin{array}{c} d_{w}=\frac{d_{w}^{0}}{s_{u}} \end{array}$$ (11)

*e*_*w*_ determines the activation time of *I*_*to*_, while the inactivation is mainly governed by *d*_*w*_. In other words, the values we adopted for *e*_*w*_ are high enough that the variable *w* can track its asymptotic value almost instantaneously, once it starts decreasing. Similarly to the *e*_1_, we defined *e*_*w*_ as:

$$\begin{array}{c} e_{w}=ge_{w}^{0} \end{array}$$ (12)

We implemented 7 by identifying the upstroke as the interval when $\frac{\partial V_{M}}{\partial t}\geq 0$ and $\frac{\partial w}{\partial t}\geq 0$. The parameter values adopted are shown in Table 1. They are optimized to reproduce experimentally measured properties of human epicardial tissue. The initial values of the variables *V*_*M*_, *u*, and *w* are set to *B* (i.e., −85 *mV*), 0, and 0, respectively.

**Table 1:** Model parameters.

*k*	*c*_1_	*c*_2_	*c*_3_	*a*	*A*	*B*	$e_{1}^{0}$	*e*_2_	*γ*_0_	*γ*_1_	*α*	*θ*_*u*_	*g*_0_	*u*_*M*_	$e_{w}^{0}$	$d_{w}^{0}$
1000 *s*^−1^	2.6	1	0.5	0.18	135 mV	-85 mV	0.0059	0.015	8	20	15	0.2	0.1	0.58	0.04	0.6

### Numerical methods

**Fig 1:**
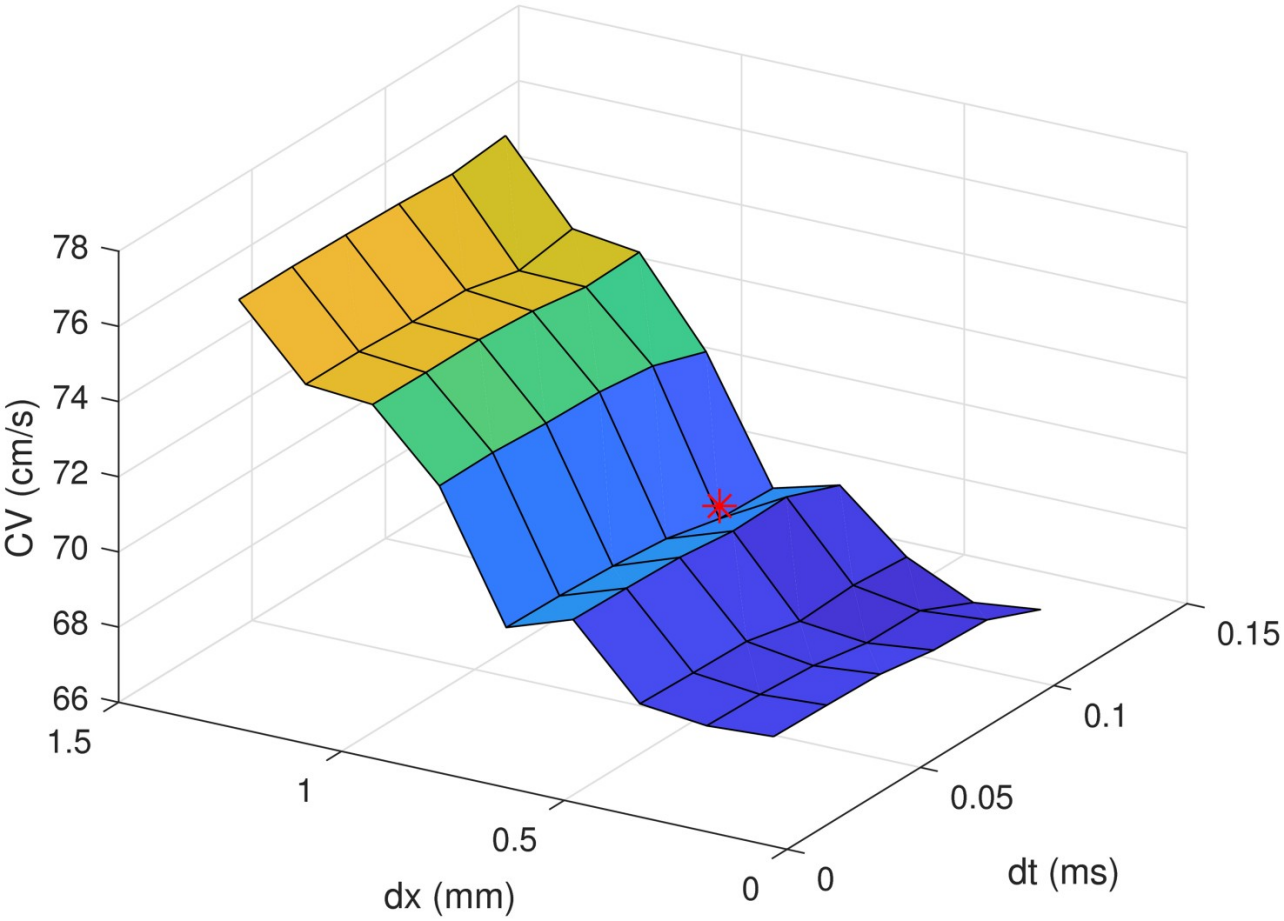
Conduction velocity for different temporal and spatial resolutions. Red marker indicates the time step and the element size adopted.

To simulate AP propagation, we incorporated our novel ionic current model in the monodomain formulation of cardiac tissue:

$$\begin{array}{c} \frac{\partial V_{M}}{\partial t}-\nabla \cdot (D\nabla V_{M})=-I_{ion}+I_{ext} \end{array}$$ (13)

where *I*_*ext*_ indicates an external stimulation current. The diffusion coefficient *D* was set to $1.171\frac{cm^{2}}{s}$. This value is directly derived in [15] from experimental values obtained in human ventricular tissue. We employed the model to perform one- and two-dimensional simulations in Comsol Multyphysics (version 5.4, COMSOL AB, Stockhlom, Sweden). The third order backward difference method was used for temporal integration with a time step of 0.1 *ms*. We selected implicit temporal integration to improve numerical stability and accuracy at larger time steps. The spatial integration was performed with the finite element method (FEM). Based on previous numerical studies [19, 26, 27], we employed third order Lagrange elements. We set the maximum element size to 0.75 *mm*, corresponding to a distance of 0.25 *mm* between nodes. For the two-dimensional simulation we used a triangular mesh. We verified the numerical accuracy of our method by evaluating the CV in a 2 *cm* long cable for different time and space resolutions (Fig 1). As previously reported for other models [28], the selection of the space step is more critical than time step selection. However, we also considered the accuracy of propagating AP morphology both in time and space, which are more evidently affected by temporal resolution. The time and space integration steps adopted produce results similar to the ones obtained with finer resolutions. Indeed, decreasing the integration steps by a factor of two results in a relative CV change of 0.96%. It is well accepted in the literature that a change of less than 5% verifies the accuracy of the numerical method [7, 8, 15]. Numerical simulations were carried out on a personal computer equipped with an Intel Core i7–3770K.

### One-dimensional simulations

One-dimensional simulations were performed on a 2 *cm* long cable. The cable was stimulated at one end with a 2 *ms* stimulus of strength twice the diastolic threshold. We calculated restitution properties with both the S1-S2 and the steady-state (or dynamic) restitution protocols. In the S1-S2 protocol, 10 S1 stimuli were applied with a cycle length of 1 *s* to reach a steady state condition. Thereafter, a S2 extrastimulus was delivered at different time intervals after the AP generated by the last S1 stimulus. At each S2 stimulation we recorded the APD, the DI, and the CV in the middle of the cable. The DI, APD pairs were used to construct the S1–S2 action potential duration restitution curve. The DI, CV pairs result in the S1-S2 conduction velocity restitution curve. The steady-state restitution protocol consists of a series of stimuli at a fixed cycle length until steady-state is reached. The cycle length was initially fixed to 1.2 *s*, and then monotonically decreased up to 2:1 conduction block. At each cycle length, the APD, the CV, and the DI were measured on the last beat, in the middle of the cable. We found that 6 beats are enough to achieve steady-state APDs and CVs. Restitution properties were computed in the middle of the cable to minimize the effects of the stimulation currents and boundaries [29]. The beginning and end of action potentials were measured using the voltage threshold corresponding to 90% repolarization (i.e., −72.5 *mV*).

### Two-dimensional simulations

We employed the S1-S2 protocol [8, 9, 30] to initiate spiral waves in a 2D sheet of ventricular epicardial tissue. The tissue size was 10 × 10 *cm*, which is large enough to prevent the boundaries from modifying the dynamic of the spiral waves. First, a single S1 stimulus was applied along the entire length of the left side of the tissue, producing a planar wave front propagating in one direction. Then, a S2 stimulus was applied at the middle of the medium in the refractory tail of the planar wave generated by S1. The S2 stimulus is applied over a rectangular region parallel to the S1 wave front but only over 80% of the edge of the tissue. The S2 wave front curls around its free end, producing a spiral wave. Both S1 and S2 stimuli lasted for 2 *ms*. S1 stimulus was twice the diastolic threshold, whereas S2 stimulus was three times the diastolic threshold. We obtained the period distribution in the spiral waves over 2 seconds of rotation after 2 seconds from S2 (thus once the spiral wave is in the steady state). Periods were measured to the nearest 1 *ms* for all points in the cardiac sheet. The trajectory of the spiral tip was traced with the zero-normal-velocity method [16]. It consists of detecting the spiral tip as the intersection of an isopotential line (*V*_*M*_ = −60 *mV* in our case) and the line $\frac{\partial V_{M}}{\partial t}=0$. Indeed, the spiral tip is defined as the point where excitation wave front and repolarization wave back meet. We also simulated the electrograms generated by the 2D tissue sheet. The electrogram was recorded with a single electrode placed at the center of the tissue sheet (0.1 mm out-of-plane). First, we considered the dipole source density in the medium as seen by the bulk. In the bidomain method, the dipole source density seen by the bulk is −*σ*_*i*_∇*V*_*M*_ [31], where *σ*_*i*_ is the intracellular conductivity. The potential in an arbitrary point can then be computed by solving the Poisson equation. By assuming infinite and homogeneous volume conductor the solution is given by [32]:

$$\begin{array}{c} V=\frac{1}{4\pi \sigma_{b}}\int \frac{-\sigma_{i}\nabla V_{M}\cdot r}{{∥r∥}^{3}}dV=\beta \int \frac{-\nabla V_{M}\cdot r}{{∥r∥}^{3}}dV \end{array}$$ (14)

where *β* is an adimensional constant. *r* is defined as the distance vector from the source point to the recording point. Since we considered two-dimensional tissues our electrograms are expressed in *mV*/*mm*.

## Results and discussion

### Action potential in tissue

**Fig 2:**
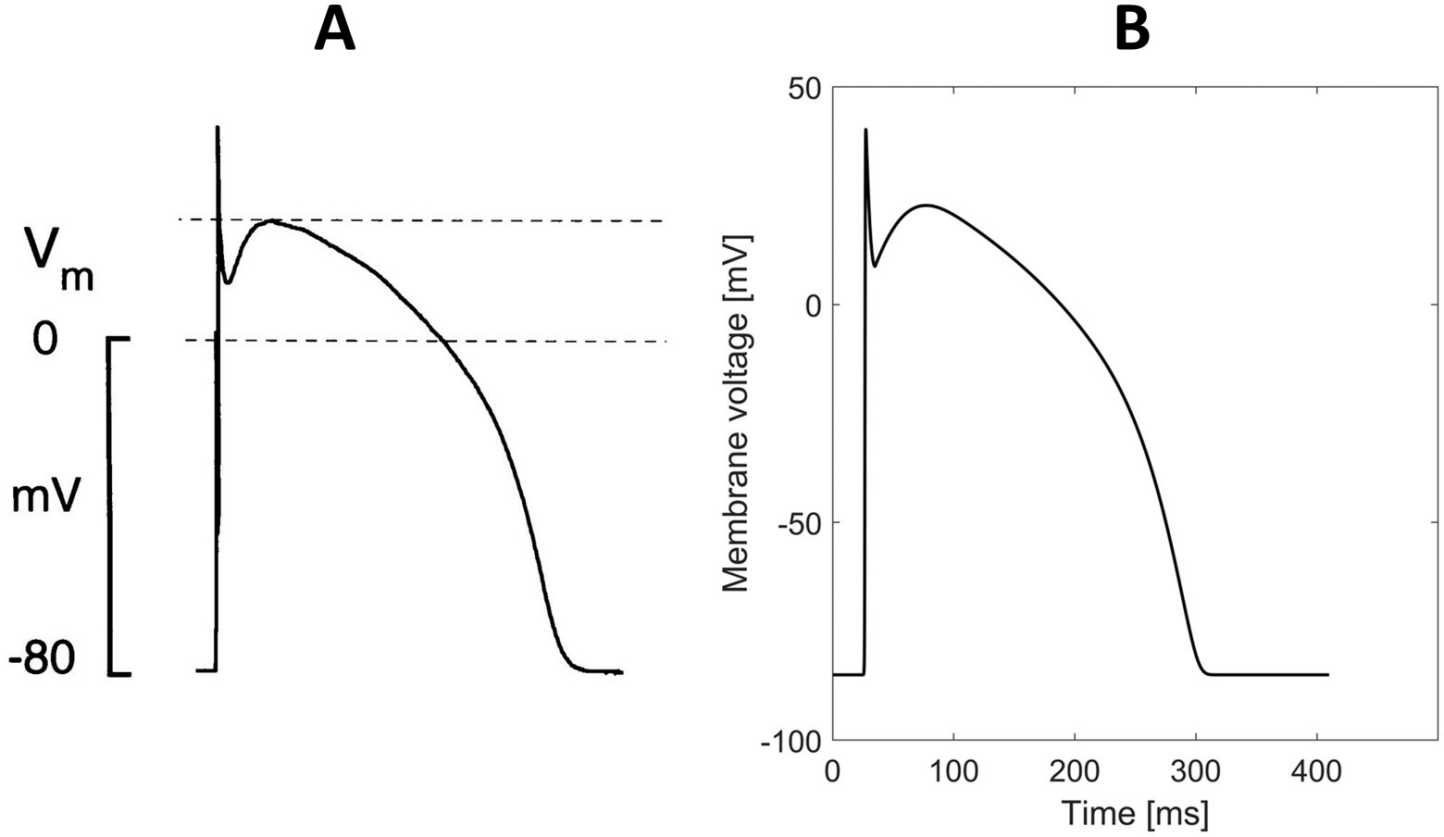
Comparison between simulated and experimental action potential. (A) Experimental action potential measured in human cardiac tissue by Nabauer et al. [20]. (B) Simulated action potential in one-dimensional cable.

Fig 2 shows the AP computed in the one-dimensional cable compared to experimental AP measured in human cardiac tissue by Nabauer et al. [20]. The maximum upstroke velocity in tissue of our model is 203 *V*/*s*, which is very close to the measurement of 196±20 *V*/*s* from Pereon et al. [21], and also close to the value of 228±11*V*/*s* measured by Drouin et al. [22]. The AP amplitude is 125 *mV*, which matches the experimental values of 123 *mV* [20] and 131 *mV* [22]. The minimum voltage of the AP notch is 8.8 *mV*, which is the mean value of experimental measurements of 8.6 *mV* [20] and 9 *mV* [23]. The maximum plateau voltage after the notch at phase 1 is 22.8 *mV*, which is very close to the experimental value of 23.7±3.1 *mV* [20]. The *APD*_90_ at 1 *Hz* cycle length is 267.1 *ms* comparable to the experimental value of 271±13 *ms* obtained by Li et al. [23]. The resting membrane potential (i.e., *B*) was set to −85 *mV*, which was in the range of experimental measurements [20–23, 33]. The excitation threshold of our model is given by *aA* + *B* = −60.7 *mV*, which matches the initiation of inward currents in human ventricular cells, occurring at approximately −60 *mV* [25]. Finally, the loss of action potential amplitude due to electrotonic currents in tissue is 2.8%, similarly to the MV model (3.1%). Indeed, the maximum voltage during upstroke occurs above the maximum plateau voltage, in agreement with experimental observations [33, 34]. Nevertheless, other physiological ionic models reproduces APs in tissue with the maximum voltage during upstroke lower than the plateau voltage [15].

**Fig 3:**
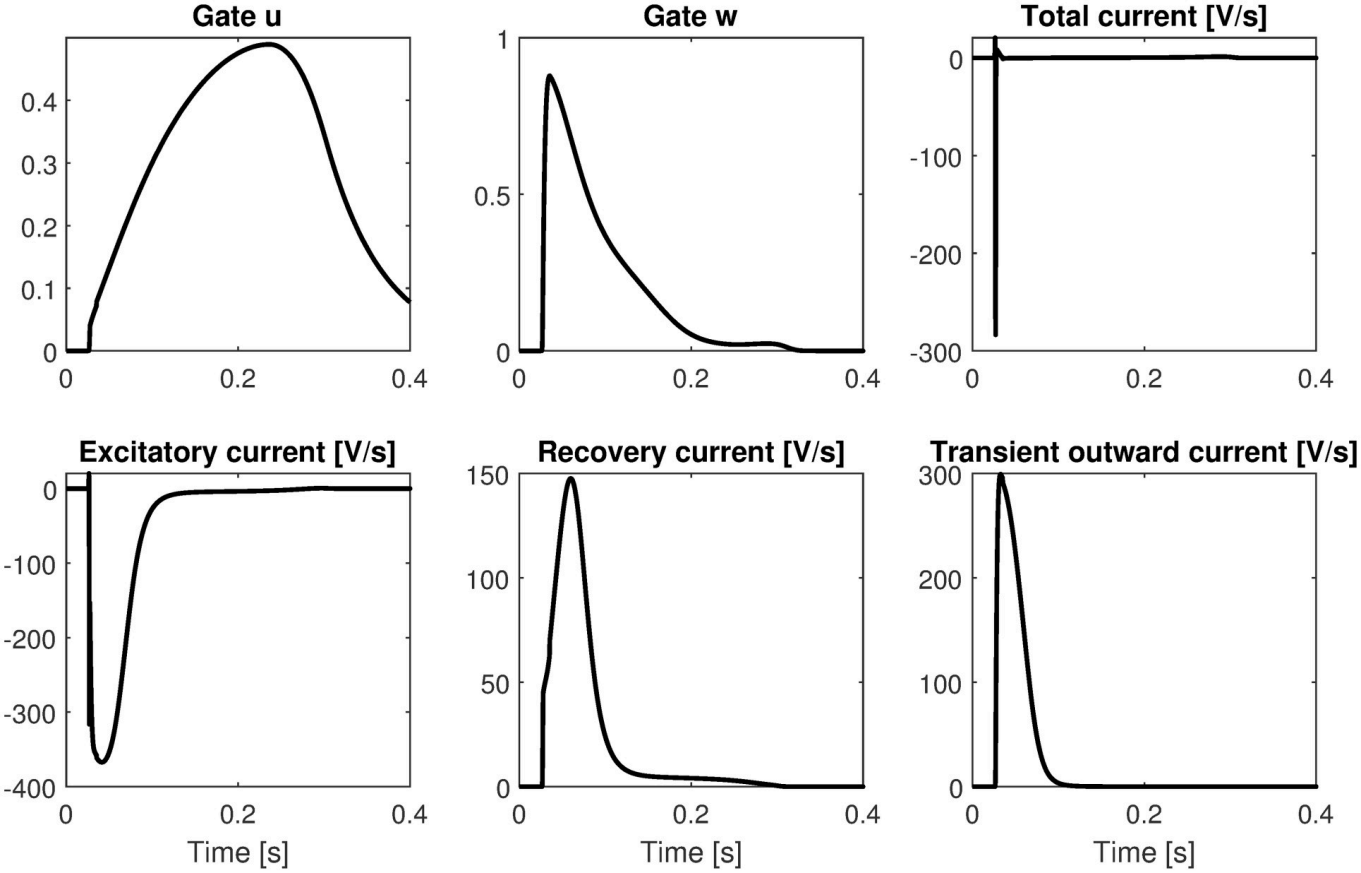
Time course of the gate variables and the ionic currents in one-dimensional cable. First row shows the time course of the two gate variable and the total ionic current. Second row shows the three contributions to the total ionic current.

Fig 3 shows the time course of the gate variables and the currents measured at the center of the one-dimensional cable. Note that the three contributions to the total current (shown in the second row) are not intended to physiologically reproduce the related ionic currents, but rather to generate a physiologically plausible total ionic current.

### Restitution properties

**Fig 4:**
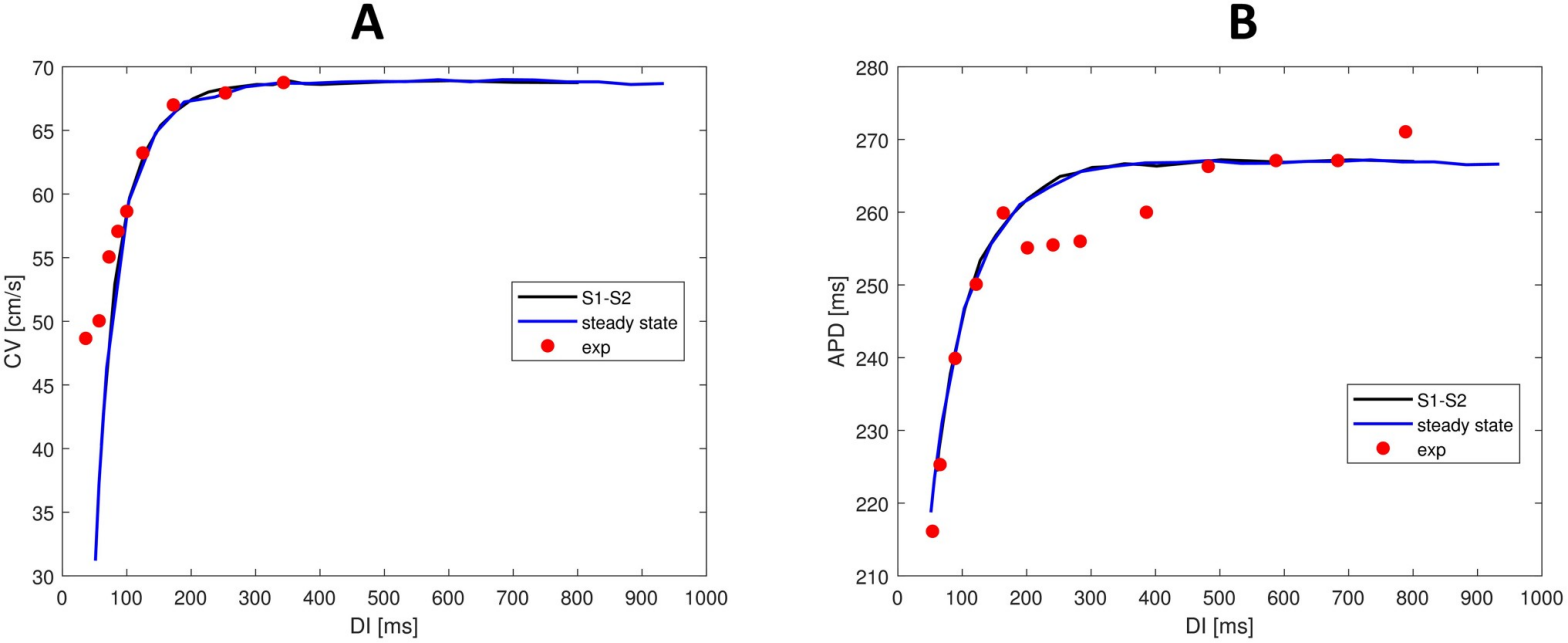
APD and CV restituition curves in tissue for the proposed model. (A) S1-S2 and steady-state APD restitution curves reproduced by our model. For comparison, experimental data from human tissue measured by Morgan et al. [24] are shown. (B) S1-S2 and steady-state CV restitution curve reproduced by our model. Experimental guinea pig data from Girouard et al. [38] are added for comparison. As in [8, 15], the experimental data are rescaled by a factor of 0.92 to get the same maximum CV level as measured in human tissue [39].

Fig 4A shows the APD restitution curves obtained with the S1-S2 and the steady-state restitution protocol, together with experimental data points from Morgan et al. [24]. Our model is able to accurately reproduce the experimental restitution curve. According to the experimental data we considered, the APD restitution curve becomes relatively flat at cycle lengths higher than 300 *ms*. However, also non-flat restitution curves, typically measured in endocardial tissue [35, 36], can be obtained with our model by making *e*_2_ linearly dependent from *u*. The slope of the APD restitution curve is always lower than one with a maximum of 0.85, comparable to experimental data from Nash et al. [37] measured in ventricular epicardium. They found a median value of 0.91 for the maximum S1–S2 (*S*1 = 600 *ms*) restitution curve slope in human epicardium, with values most frequently in the range 0.5–1.

Fig 4B shows the CV restitution curves obtained with the S1-S2 and the steady-state restitution protocols. Due to the absence of detailed and reliable human ventricular CV restitution data, we employed an experimental guinea pig CV restitution curve for comparison [38]. As in [8, 15], the guinea pig data were rescaled by a factor of 0.92 to match the maximum CV of about 70 *cm*/*s* measured in human tissue [39]. From steady-state restitution curves we also obtained the minimum cycle length for propagation, which is equal to 270 *ms*. The corresponding minimum DI is 51 *ms*, while the minimum APD is 219 *ms*. Maximum and minimum CVs are 69 *cm*/*s* and 31 *cm*/*s*, respectively. Consequently, CV dispersion is equal to 38 *cm*/*s* (i.e., 55%), that matches experimental dispersion of 55% measured by Nanthakumar et al. [40].

The steady-state and the S1-S2 restitution curves are almost identical, suggesting absence of memory effects. Indeed, refractoriness in our model is uniquely determined by the variable *u*. Therefore, the restitution properties of the model only depends on the DI and not on previous pacing cycle length.

### Spiral waves

**Fig 5:**
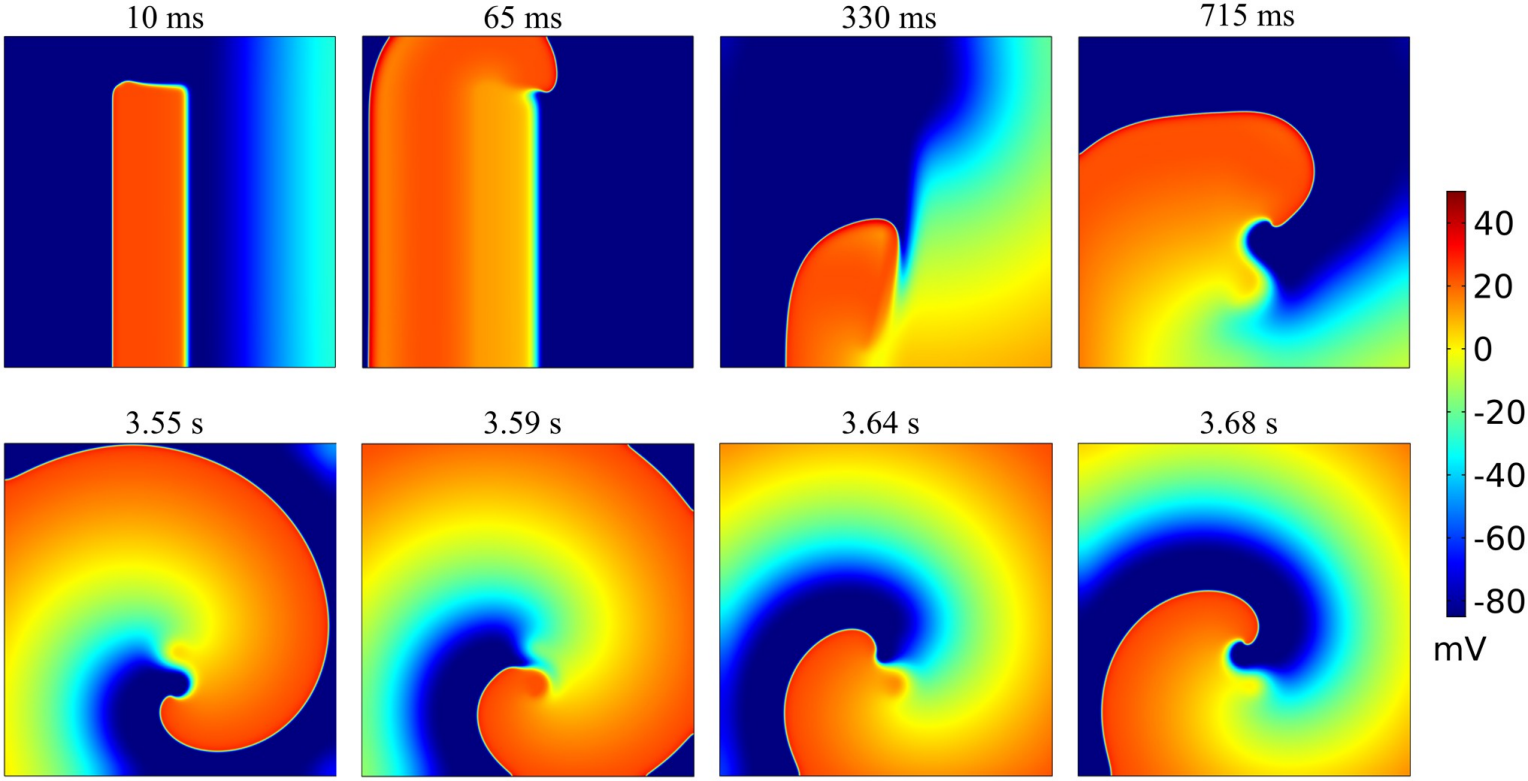
Reentrant spiral wave dynamics in 2D reproduced by our model. First row shows snapshots of the membrane potential during spiral wave initiation. Second row shows steady-state dynamics over an half period. Time instants above the pictures are relative to the delivery of S2 stimulus.

**Fig 6:**
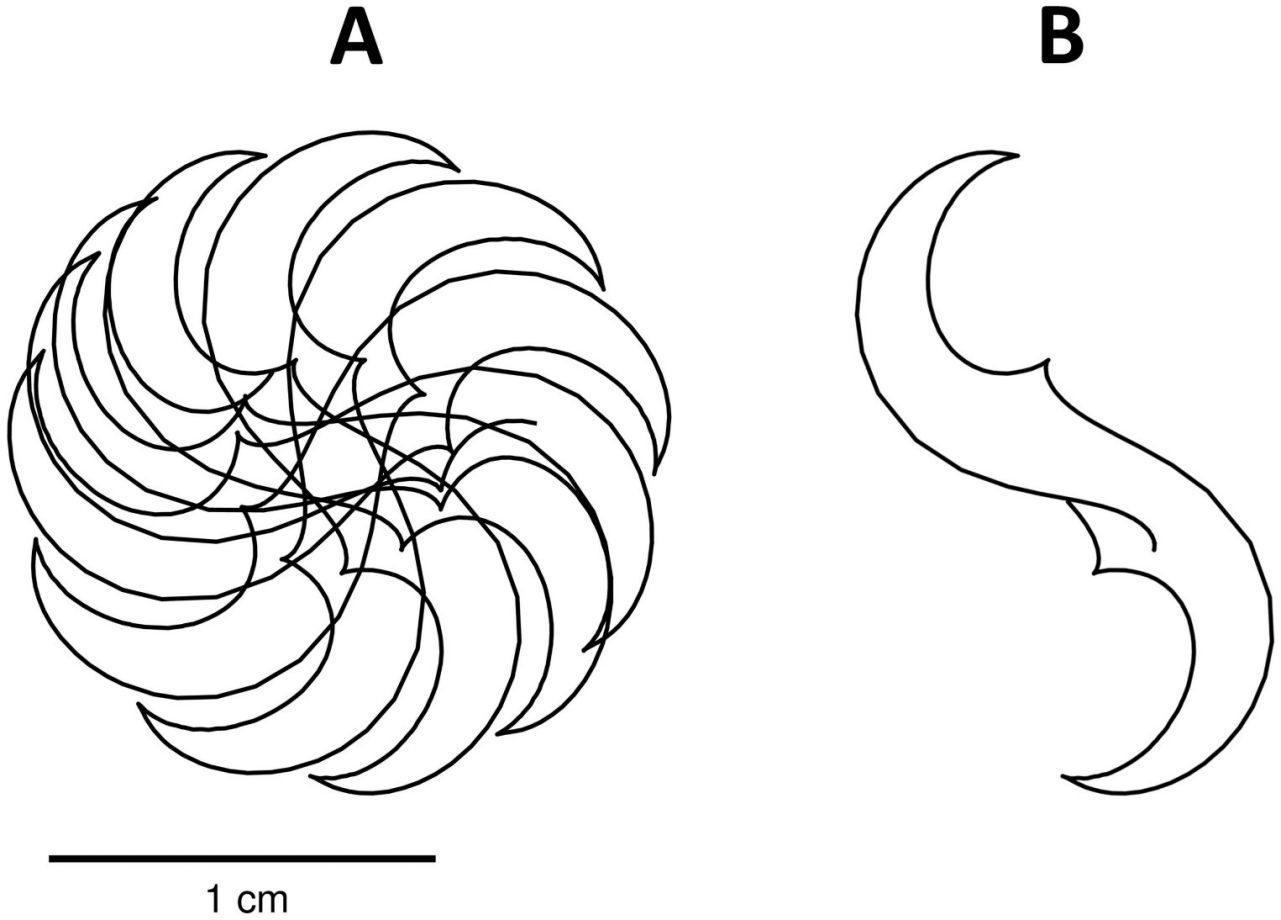
Spiral tip trajectory during reentry dynamics. (A) Spiral tip trajectory over 1.5 *s* of simulation after 2.5 *s* the S2 stimulus. (B) Spiral tip trajectory over a single full rotation.

Fig 5 shows initiation (first row) and steady-state dynamics (second row) of spiral waves reproduced by our model. The S2 stimulus generates an activation at the center-left of the tissue, but only for the 80% of the height. The wave curls around its free end, generating a spiral wave. After some transient rotations, the spiral wave stabilizes (see S1 Video). The reentry dynamics is characterized by a slightly meandering core (Fig 6), very similar to TNNP dynamics. Indeed, the tip trajectory for a single rotation traces an S-shaped core. Similarly to the Z core of the TNNP model, it combines regions of fast rotation of the tip, typical of a circular core, with regions of laminar motion typical of a linear core. The spiral core has cross-section of about 2 *cm*, which is lower than in TNNP and MV models (∼3 *cm*), probably due to the lower upstroke velocity of our model.

**Fig 7:**
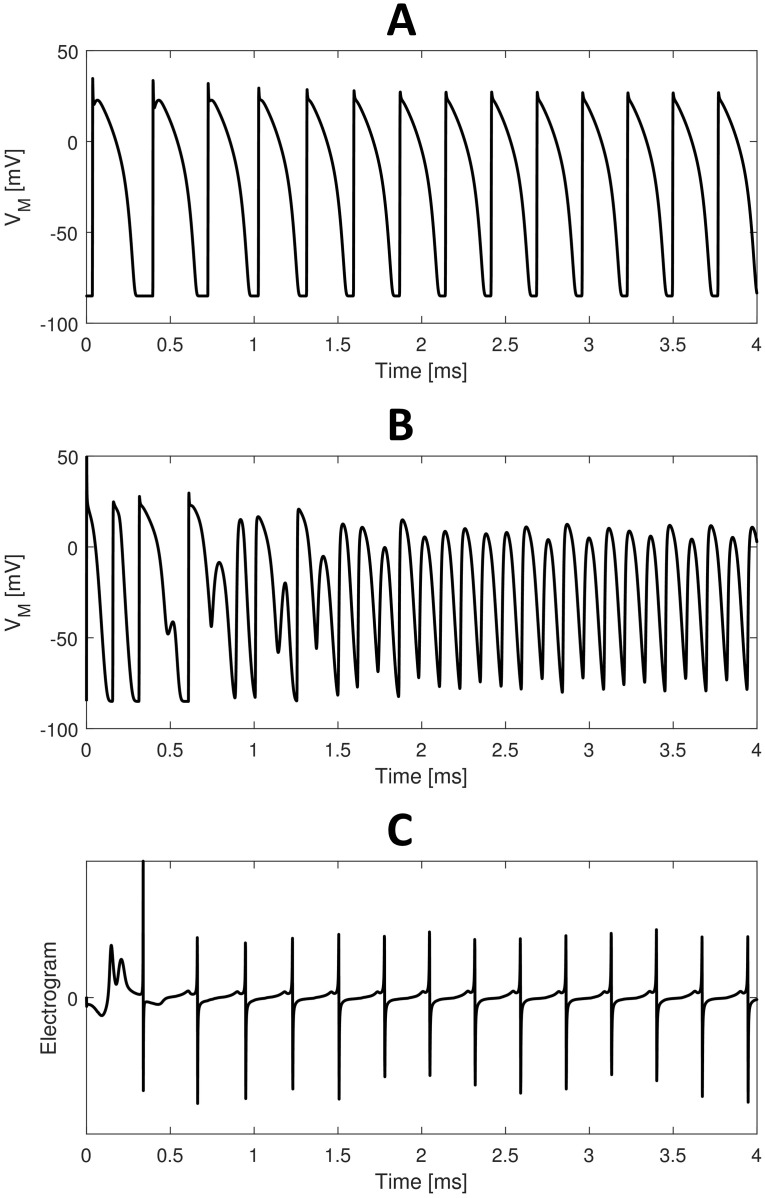
Membrane potentials and electrograms during reentry dynamics. (A) Membrane potential recorded in a point far from the tip (asterisk in Fig 8A). (B) Membrane potential recorded at the center of the spiral core (circle in Fig 8A). (C) Simulated electrogram. Time axis indicates time after S2 stimulus delivery.

Fig 7 shows the membrane action potential in a point far from the tip and in the center of the spiral core, computed as the middle point of the spiral tip trajectory. Far from the tip (Fig 7A) the membrane potential is highly regular with relatively long APs. In the center of the spiral core (Fig 7B) the membrane potential is irregular, especially during the initiation of the spiral wave, and APs are much shorter. Indeed, the center of the spiral core experiences continuous conduction block. Therefore, electrotonic coupling with refractive surrounding tissue generates very short APs.

**Fig 8:**
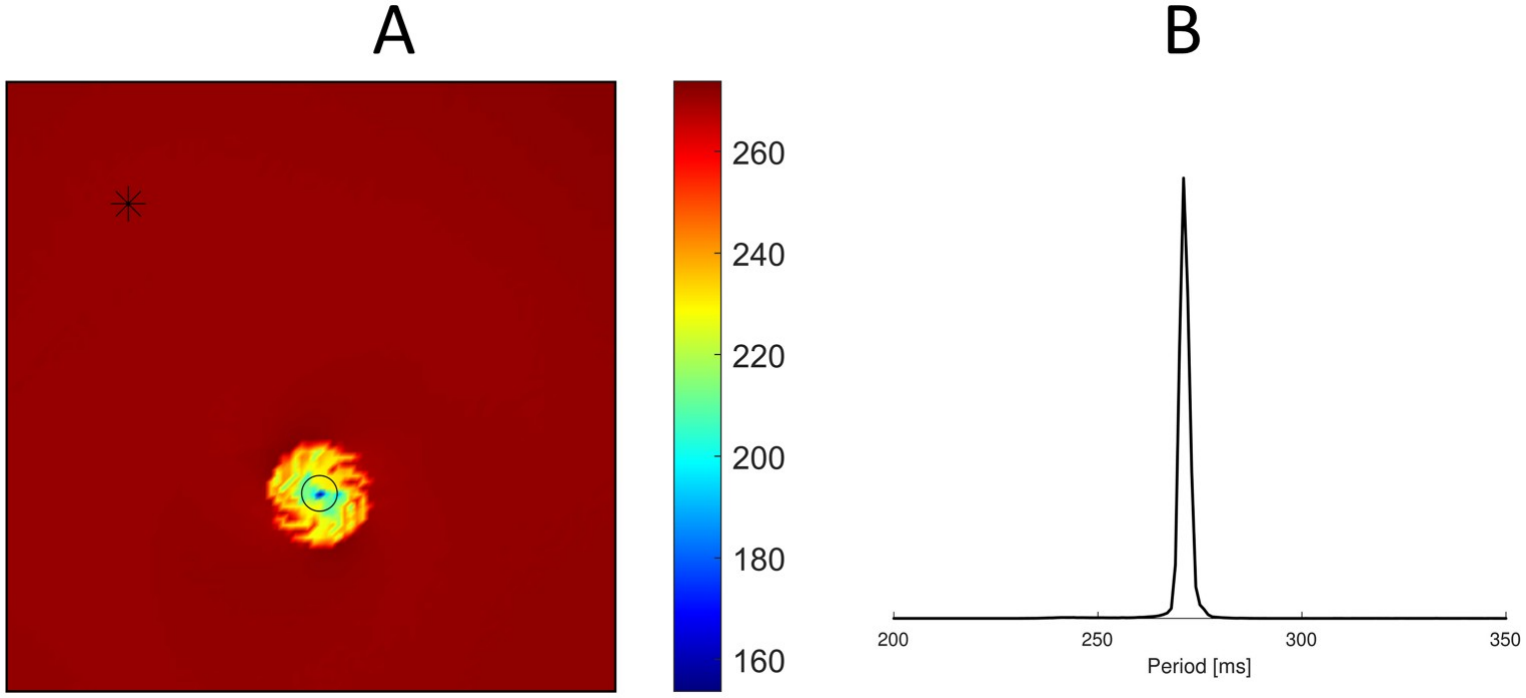
Mean period map and period spectra during reentry dynamics. (A) Mean period map computed over 2 *s* of simulation after 2 *s* the S2 stimulus. The black markers indicates the two point where membrane potentials of Fig 7 were evaluated. (B) Period spectra over 2 *s* of simulation after 2 *s* the S2 stimulus.

Fig 7C shows the electrogram recorded at the center of the tissue sheet. After stabilization of the spiral wave, the electrogram becomes regular with a pattern similar to clinical epicardial electrograms during ventricular tachycardia [41, 42]. We also computed the mean period between subsequent activations for each node. The mean period map (Fig 8A) shows an almost constant period everywhere, except for the spiral core. As expected, traces of the spiral core are characterized by shorter APs. Minimum APD was obtained at the center of the spiral core. These findings suggest that the mean period map could be used to roughly estimate the spiral tip trajectory. Finally, the period spectra (Fig 8B) shows a dominant period of 271 *ms* corresponding to a frequency of 3.69 *Hz*. This finding matches the experimental results from Koller et al. [36] who recorded dominant periods during ventricular tachycardia ranging between 246 *ms* and 352 *ms*.

### Computational efficiency

An important characteristic of our model is the computational efficiency and the suitability to perform large-scale simulations. To assess the computational efficiency of our model with respect to the available literature, we implemented a single cell simulation by using both our model and the MV model. We selected the MV model for the comparison for two reasons. First, it is the first phenomenological model reproducing the tissue-level characteristics of human myocytes, and is based only on four variables. Second, the computational complexity of the minimal model was already compared to the TNNP, PB and IMW models in [15]. One hundred simulations of 10 *s* were performed to obtain a robust mean computational time for the two models. We adopted a simple forward Euler scheme with a time step of 0.01 *ms* (as used in [15]) for both the models to avoid the results being affected by the integration method. Results show that the proposed model is about two times faster than the MV model. In particular, on an Intel Core i7–3770K a simulation of 10 *s* requires on average about 0.21 *s* for the MV model and 0.12 *s* for our model.

## Conclusion

We have presented a novel model for human epicardial tissue derived from the Rogers-McCulloch formulation of the FHN model. The model represents the total ionic current divided into three contributions corresponding to the excitatory, recovery, and transient outward currents. Our model reproduces the main characteristics of human epicardial tissue, including AP amplitude and morphology, upstroke velocity, and APD and CV restitution curves. The main advantage of our model is its simple formulation, which is determined by only 3 variables and 17 parameters. In contrast, the MV model employs 4 variables and 30 parameters. We found that our model is almost two times faster than the MV model. The low number of parameters allow easy understanding of the role of each parameter in determining the dynamical behaviour of the system and facilitates the process of fitting the model to the experimental data.

In future, to further reduce the computational power required and to facilitate three-dimensional large-scale simulations, we could simplify our model following the approach suggested by Bernus et al. in [43, 44]. The approach consists in slowing down the dynamics during the upstroke to allow reduction of the integration steps, while increasing the diffusivity to maintain the original conduction velocity. The result of the simplification is characterized by a longer upstroke, similarly to highly simplified models such as the FHN [18, 19] and Aliev-Panfilov [30] models.

Complex physiological models consists of a large number of variables, varying from 17 (TNNP model) to 67 (IMW model). Therefore, their applicability can be limited by their intense computational demands. As consequence, most of such studies show only single cell simulations (e.g., [12, 13]). Moreover, the large number of parameters makes it extremely difficult to understand the influence of the single parameters on the dynamics of the model. Thus, also the fitting process becomes more challenging. Nevertheless, biophysically detailed models are necessary to study certain types of phenomena, such as the role of ionic concentrations changes and single ionic channels. We believe that the physiological and phenomenological models are both important tools in the study of the electrical activity of the heart with different application fields. Physiological models should be selected for studies regarding the behaviour of single myocytes and single ionic channels. On the other hand, phenomenological models are more suitable for large-scale 2D and 3D simulations where the use of biophysically detailed models could be computationally challenging.

We employed the proposed model to reproduce only epicardial tissue. However, we believe that our model could be fitted also to the other types of ventricular cells (i.e. endocardial and midcardial), as well as to atrial cells.

In future studies, we plan to employ our model to investigate reentry phenomena induced by pathological conditions, such as cardiac ischemia (e.g. [45, 46]) and Brugada syndrome (e.g. [47, 48]). We also plan to investigate the ability of our model to reproduce alternans. Indeed, with the actual parameters set the model cannot reproduce alternans. However, alternans could be obtained by increasing the value of *e*_2_. This modification would reduce the minimum DI and makes the S1-S2 APD restitution curve steeper. Indeed, the ability to reproduce alternans has been linked to the steepness of the APD restitution curves, even if other factors, such as CV restitution curve, electrotonic interactions, and cardiac memory have shown to play a role in generating instabilities [9, 49].

## Supporting information

S1 Raw image (JPG)

S1 Video Animation of the spiral wave. Time is relative to the delivery of the S2 stimulus. After the first 2 seconds of the simulation, a trace of the spiral tip trajectory relative to the last 200 *ms* is also shown. Color bar unit is V. Axes unit is meter.
(MP4)
