# Supplementary figures and images for: A computationally efficient dynamic model of human epicardial tissue
Source: PLoS One. 2021 Oct 26;16(10):e0259066. doi: 10.1371/journal.pone.0259066 (PMC8547700; doi:10.1371/journal.pone.0259066)

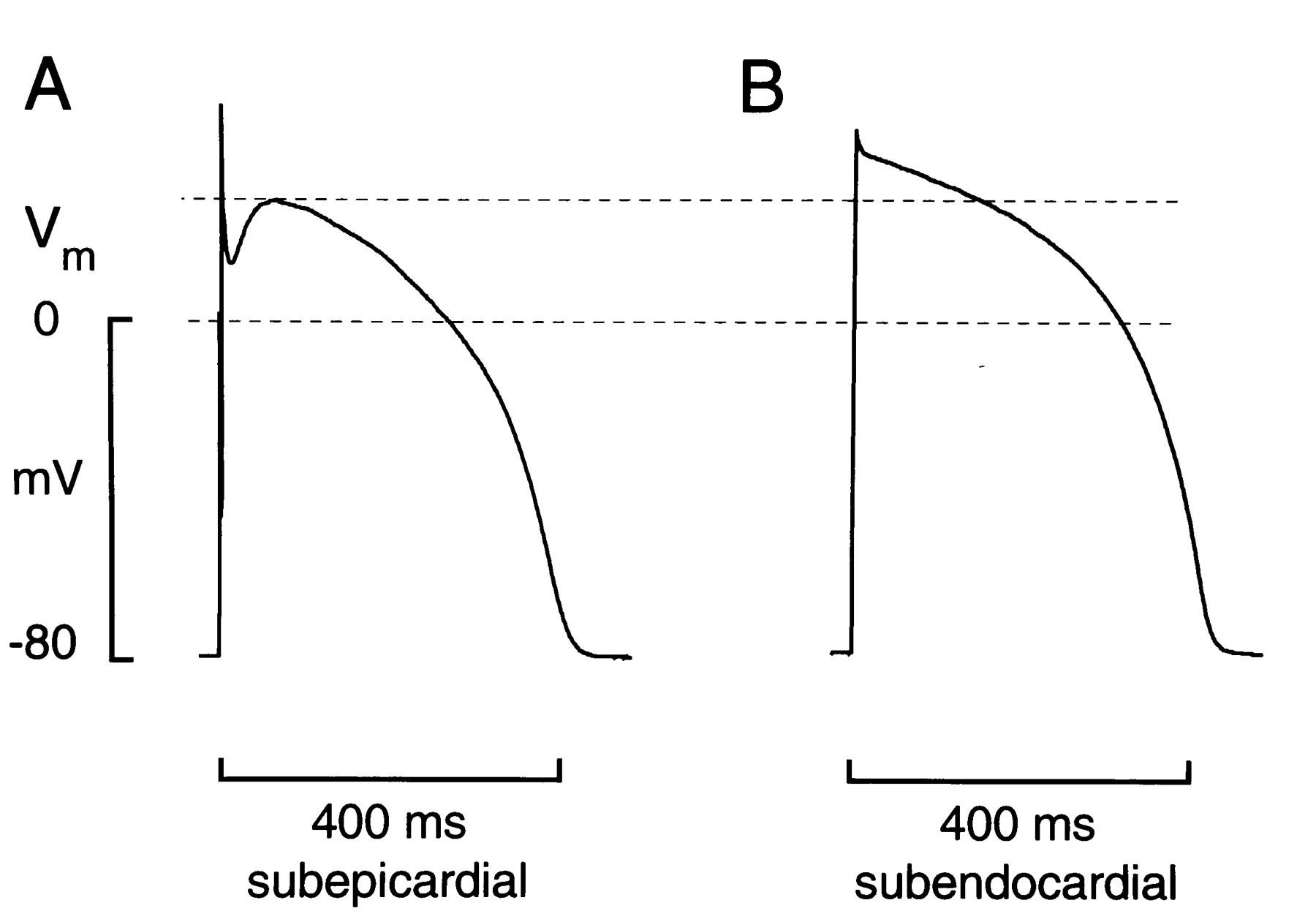

Supplement: S1 Raw image — (JPG) [file pone.0259066.s001.jpg]
